# Diagnostic Value of Multidetector CT and Its Multiplanar Reformation, Volume Rendering and Virtual Bronchoscopy Postprocessing Techniques for Primary Trachea and Main Bronchus Tumors

**DOI:** 10.1371/journal.pone.0137329

**Published:** 2015-09-02

**Authors:** Mingyue Luo, Chaijie Duan, Jianping Qiu, Wenru Li, Dongyun Zhu, Wenli Cai

**Affiliations:** 1 Department of Radiology, The Sixth Affiliated Hospital of Sun Yat-sen University, Guangzhou, Guangdong, China; 2 Research Center of Biomedical Engineering, Graduate School at Shenzhen, Tsinghua University, Shenzhen, Guangdong, China; 3 Department of Radiology, Massachusetts General Hospital and Harvard Medical School, Boston, Massachusetts, United States of America; University of Nebraska Medical Center, UNITED STATES

## Abstract

**Purpose:**

To evaluate the diagnostic value of multidetector CT (MDCT) and its multiplanar reformation (MPR), volume rendering (VR) and virtual bronchoscopy (VB) postprocessing techniques for primary trachea and main bronchus tumors.

**Methods:**

Detection results of 31 primary trachea and main bronchus tumors with MDCT and its MPR, VR and VB postprocessing techniques, were analyzed retrospectively with regard to tumor locations, tumor morphologies, extramural invasions of tumors, longitudinal involvements of tumors, morphologies and extents of luminal stenoses, distances between main bronchus tumors and trachea carinae, and internal features of tumors. The detection results were compared with that of surgery and pathology.

**Results:**

Detection results with MDCT and its MPR, VR and VB were consistent with that of surgery and pathology, included tumor locations (tracheae, n = 19; right main bronchi, n = 6; left main bronchi, n = 6), tumor morphologies (endoluminal nodes with narrow bases, n = 2; endoluminal nodes with wide bases, n = 13; both intraluminal and extraluminal masses, n = 16), extramural invasions of tumors (brokethrough only serous membrane, n = 1; 4.0 mm—56.0 mm, n = 14; no clear border with right atelectasis, n = 1), longitudinal involvements of tumors (3.0 mm, n = 1; 5.0 mm—68.0 mm, n = 29; whole right main bronchus wall and trachea carina, n = 1), morphologies of luminal stenoses (irregular, n = 26; circular, n = 3; eccentric, n = 1; conical, n = 1) and extents (mild, n = 5; moderate, n = 7; severe, n = 19), distances between main bronchus tumors and trachea carinae (16.0 mm, n = 1; invaded trachea carina, n = 1; >20.0 mm, n = 10), and internal features of tumors (fairly homogeneous densities with rather obvious enhancements, n = 26; homogeneous density with obvious enhancement, n = 1; homogeneous density without obvious enhancement, n = 1; not enough homogeneous density with obvious enhancement, n = 1; punctate calcification with obvious enhancement, n = 1; low density without obvious enhancement, n = 1).

**Conclusion:**

MDCT and its MPR, VR and VB images have respective advantages and disadvantages. Their combination could complement to each other to accurately detect locations, natures (benignancy, malignancy or low malignancy), and quantities (extramural invasions, longitudinal involvements, extents of luminal stenoses, distances between main bronchus tumors and trachea carinae) of primary trachea and main bronchus tumors with crucial information for surgical treatment, are highly useful diagnostic methods for primary trachea and main bronchus tumors.

## Introduction

CT is the best noninvasive method for evaluation of trachea and main bronchus lesions, but the overwhelming majority of studies on detection of trachea and main bronchus tumors were confined to ordinary CT or ordinary spiral CT[1–3]. Multidetector CT (MDCT) could contribute to a better revealing of trachea and main bronchus tumors because of improved image resolution and quality[4, 5]. Moreover, multiplanar reformation (MPR), volume rendering (VR) and virtual bronchoscopy (VB) postprocessing techniques of MDCT could break away from the confines of traditional axial imaging plane, and have the potential to facilitate assessment of trachea and main bronchus tumors, providing more anatomically and diagnostically meaningful information for detection of trachea and main bronchus tumors[4–12]. Hoppe et al[12] found that MDCT VB is a reliable noninvasive method to accurate grading of tracheobronchial stenosis, but it should be combined with the interpretation of axial CT images and MPR images for evaluation of surrounding structures and optimal spatial orientation. However, up to date, their applications in primary trachea and main bronchus tumors has defect, there is no systematic and complete study on primary trachea and main bronchus tumors with MDCT and its MPR, VR and VB postprocessing techniques. Therefore, the purpose of this paper was to evaluate the diagnostic value of MDCT and its MPR, VR and VB postprocessing techniques for primary trachea and main bronchus tumors.

## Materials and Methods

### Clinical data

Thirty-one primary trachea and main bronchus tumors proved by surgery and pathology were included in this retrospective study. The tumors were composed of 1 adenoma, 1 adenoid cystic carcinoma, 14 squamous cell carcinomas, 2 adenocarcinomas, and 1 leiomyosarcoma of tracheae; 5 squamous cell carcinomas, and 1 adenocarcinoma of right main bronchi; 1 leiomyoma, 1 mucoepidermoid carcinoma, and 4 squamous cell carcinomas of left main bronchi. The consecutive patients consisted of 27 males and 4 females with age range from 28.0 to 72.0 years old (mean age, 48.6 years old) (S1 Table). Chemotherapy or radiotherapy was not performed before surgery for all patients.

As a retrospective study, its MDCT examination and image postprocessing techniques were conventional and routine practice in our Department of Radiology, thus it needed no special ethical approval. Furthermore, the data were analyzed anonymously and reported. Therefore, the ethical approval and informed consent were waived.

### MDCT examination

MDCT scanning was performed by using a LightSpeed QX/i CT scanner (General Electric Medical Systems, Milwaukee, Wis, USA). Firstly, precontrast scanning of chest was done, a detector row configuration of 2.5 mm, and a pitch of 3.0 from thoracic inlet to base of the lung. Then, all patients received contrast medium (Iopamiro, 300 mg I/ml; Schering Pharmacy, Guangzhou, China) enhancement scanning. The nonionic low osmolarity contrast medium was bolus administered by means of a power injector at a rate of 2.5 ml/sec through a 20-gauge plastic intravenous catheter placed in the antecubital vein. Volume of contrast medium delivered was 1.5 ml /kg. Scanning time delay was 25.0 and 90.0 seconds, respectively.

### Image postprocessing techniques

Image postprocessing data were transferred to a workstation (Sun Microsystems, Mountain View, Calif, USA) via picture archive and communication system after retroconstructing the scanning image data with 1.5 mm thickness, 0.5 mm interval. The workstation was equipped with commercially available postprocessing software (Advantage Windows 4.5; General Electric Medical Systems, Milwaukee, Wis, USA). MPR, VR and VB images were obtained with postprocessing techniques in the workstation.

MPR images[11, 13–15]: axial, coronal, sagittal, and oblique images were acquired section by section with centering on trachea and main bronchus tumors by using MPR software, and to display the walls, lumina, and adjacent structures of trachea and main bronchus tumors. VR images[16–18]: firstly, image reconstruction of trachea and main bronchus area was performed by making use of VR software; secondly, unnecessary parts were cut off with Scalpel program, and interested trachea and main bronchus images were obtained; thirdly, images were magnified and rotated locally to display trachea and main bronchus tumors as clear as possible. VB images[12, 19–22]: endoluminal images of trachea and main bronchus were generated by applying Navigator software with about –700 HU threshold, tumors were observed with Fly-through program along longitudinal lumina.

### Result analyses and comparison with that of surgery and pathology

Detection results of primary trachea and main bronchus tumors with MDCT and its MPR, VR and VB, were analyzed retrospectively by two highly experienced radiologists who were blind to surgical and pathological results with regard to tumor locations, tumor morphologies, extramural invasions of tumors, longitudinal involvements of tumors, morphologies and extents of luminal stenoses, distances between main bronchus tumors and trachea carinae, and internal features of tumors. If their analyses differed, the two radiologists reached a consensus reading after reviewing and discussing the controversial detection results with a senior radiologist. Detection results of 31 primary trachea and main bronchus tumors with MDCT and its MPR, VR and VB, were compared with surgical and pathological results.

## Results

### Tumor locations

One benign tumor, 1 low malignant tumor and 17 malignant tumors were located in tracheae. Six malignant tumors were located in right main bronchi. One benign tumor, 1 low malignant tumor and 4 malignant tumors were located in left main bronchi. Tumor locations were in correspondence with surgical results.

### Tumor morphologies

Two tumors displayed as endoluminal nodes with narrow bases. Thirteen tumors appeared as endoluminal nodes with wide bases. Sixteen tumors grew infiltratedly along thickened walls with stenosed lumina, and formed both intraluminal and extraluminal masses. Tumor morphologies were in accordance with results of surgery and pathology.

### Extramural invasions of tumors

Low malignant and malignant tumors grew infiltratedly along walls with different extramural invasions. One mucoepidermoid carcinoma of left main bronchus brokethrough only serous membrane with coarse margin. One squamous cell carcinoma of right main bronchus had extramural invasion with large extramural mass, had no clear border with right atelectasis. Extramural invasions of the rest 14 malignant tumors were 4.0 mm–56.0 mm. Extramural invasions of tumors were correspondent with surgical and pathological results.

### Longitudinal involvements of tumors

**Fig 1:**
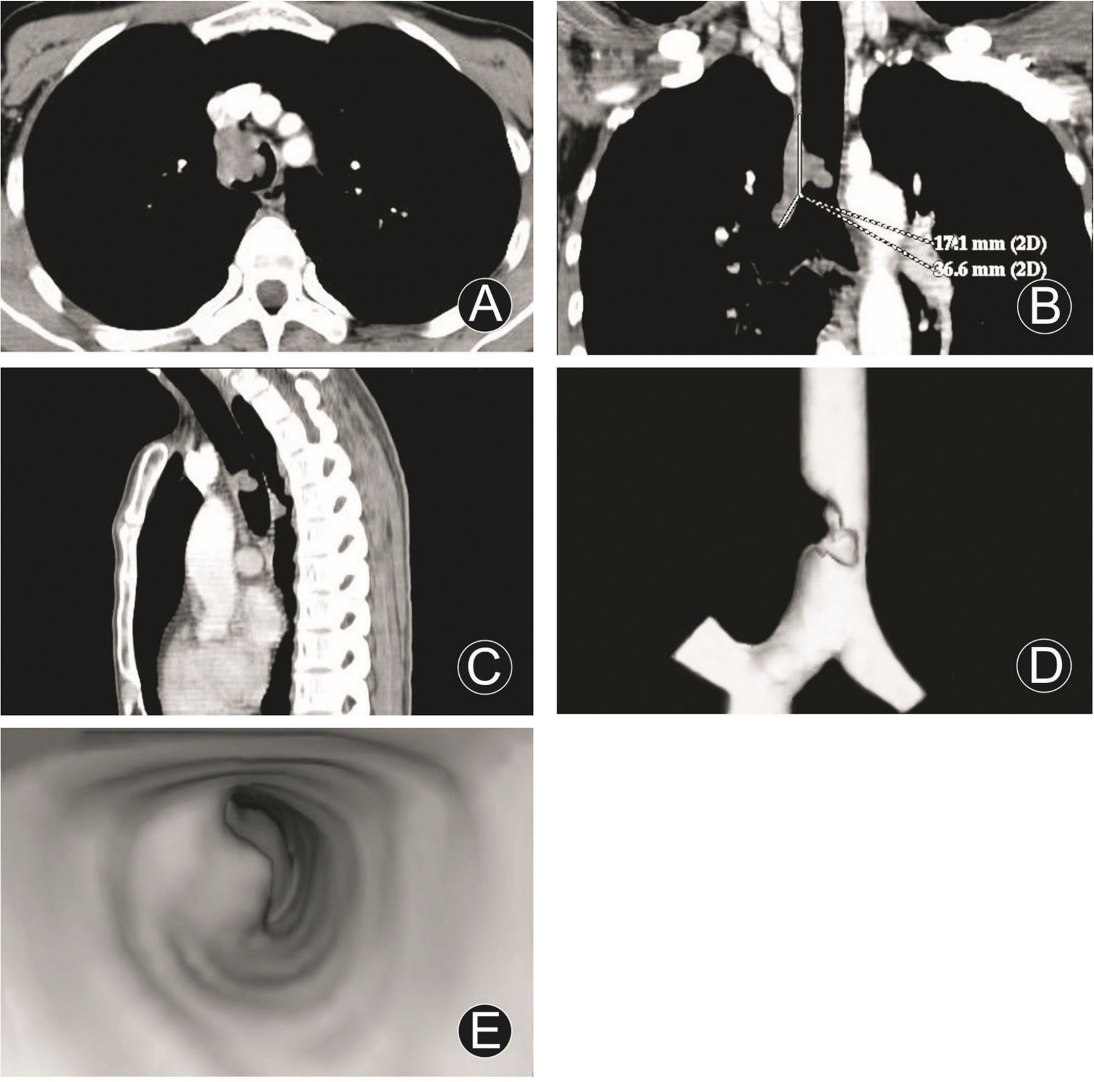
Squamous cell carcinoma of the lower trachea and involvement of right main bronchus. MDCT axial image (A), MPR (coronal, B; sagittal, C), VR (D) and VB (E) images disclosed a wide based and irregular tumor, protruded into endolumen with severe eccentric lumenial stenosis, still homogeneous density and fairly obvious enhancement, extending to right main bronchus, and longitudinal involvement of 53.7 mm.

Longitudinal involvements of tumors were different. Longitudinal involvement of endoluminal nodular tumor with narrow basis was small, longitudinal involvement of 1 adenoma with peduncle in trachea carina was only 3.0 mm. One squamous cell carcinoma of the lower trachea with involvement of right main bronchus and invasion extraluminally, its longitudinal involvement was 53.7 mm (Fig 1). One squamous cell carcinoma of right main bronchus involved the whole right main bronchus wall and trachea carina. Longitudinal involvements of the remainder 28 tumors were 5.0 mm–68.0 mm. They were consistent with that of surgery and pathology.

### Morphologies and extents of luminal stenoses

Morphologies of luminal stenoses included irregular (n = 26, 83.8%), circular (n = 3, 9.6%), eccentric (n = 1, 3.3%), and conical (n = 1, 3.3%). Extents of luminal stenoses were composed of mild (extent, ≤25%; n = 5, 16.1%), moderate (extent, 26%–75%; n = 7, 22.6%), and severe (extent, ≥76%; n = 19, 61.3%). They consisted with surgical results.

### Distances between main bronchus tumors and trachea carinae

Distance between 1 squamous cell carcinoma of left main bronchus and trachea carina was 16.0 mm. One squamous cell carcinoma of right main bronchus not only invaded the whole right main bronchus wall but also involved trachea carina. Distances between the rest 10 main bronchus tumors and trachea carinas were >20.0 mm. They coincided with that of surgery.

Comparison between detection results with MDCT and its MPR, VR and VB and surgical and pathological results about above 6 items was summarized in Table 1.

**Table 1:** Comparison between detection results with MDCT and its MPR, VR and VB and surgical and pathological results of 31 primary trachea and main bronchus tumors.

Detection item with MDCT and its MPR, VR and VB	Compared with surgical and pathological results
Tumor locations	In correspondence with surgical results
Tumor morphologies	In accordance with surgical and pathological results
Extramural invasions of tumors	Correspondent with surgical and pathological results
Longitudinal involvements of tumors	Consistent with surgical and pathological results
Morphologies of luminal stenoses	Consisted with surgical results
Extents of luminal stenoses	Consisted with surgical results
Distances between main bronchus tumors and trachea carinae	Coincided with surgical results

### Internal features of tumors

They were disclosed only with MDCT and its MPR images, VR and VB images could not do it. One leiomyosarcoma of trachea had not enough homogeneous density with obvious enhancement, 1 adenoid cystic carcinoma of trachea had low density without obvious enhancement. One leiomyoma of left main bronchus had homogeneous density and obvious enhancement, 1 mucoepidermoid carcinoma of left main bronchus had punctuate calcification with obvious enhancement. One adenoma in trachea carina had homogeneous density without obvious enhancement. Twenty-three squamous cell carcinomas and 3 adenocarcinomas had fairly homogeneous densities and rather obvious enhancements (S1 Table).

### Findings of benign and malignant tumors

**Fig 2:**
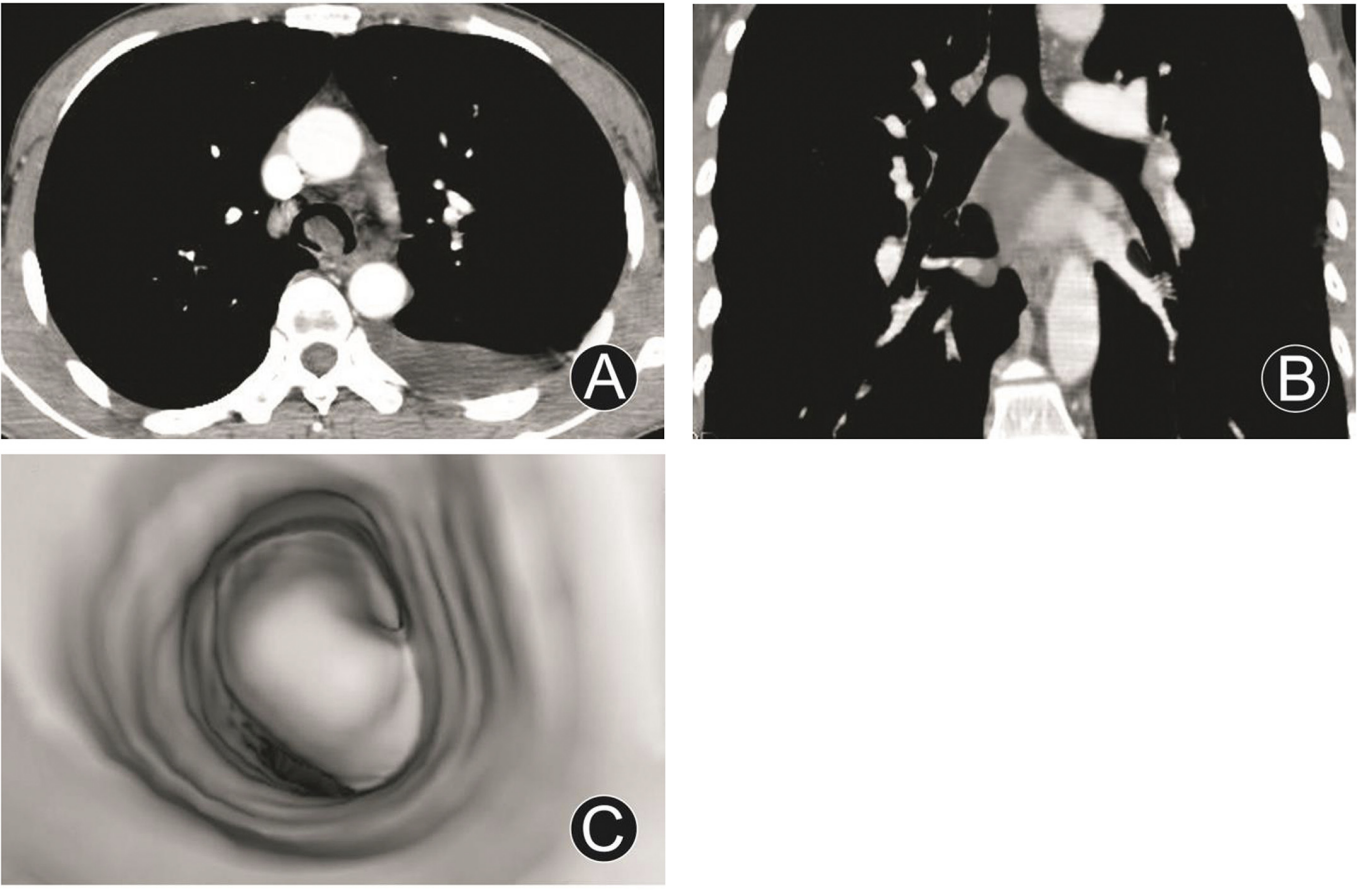
Adenoma in trachea carina. MDCT axial image (A), MPR (oblique, B) and VB (C) images revealed around and polypiform mass, and protruded into endolumen with a narrow peduncle connected to wall, not thickened wall, severe eccentric lumenial stenosis, and homogeneous density without obvious enhancement.

One adenoma in trachea carina appeared as round and polypiform mass, and protruded into endolumen with a narrow peduncle connected to wall (Fig 2); one leiomyoma of left main bronchus disclosed as oval and polypiform mass, and protruded into endolumen; they both had homogeneous densities with no obvious enhancements and thickened walls. One adenoid cystic carcinoma of trachea displayed as polypiform mass and protruded into endolumen with a wide base, homogeneous density, and fairly smooth and regular margin. One mucoepidermoid carcinoma of left main bronchus grew both intraluminally and extraluminally with slightly thickened wall and coarse serous membrane. Twenty-three squamous cell carcinomas, 3 adenocarcinomas, and 1 leiomyosarcoma showed as irregular or cayliflower masses and protruded into endolumen with irregular luminal stenoses, infiltrated and thickened walls; squamous cell carcinomas and adenocarcinomas had homogeneous densities and quite obvious enhancements, leiomyosarcoma had not enough homogeneous density and obvious enhancement; their size were 28.0 mm–94.0 mm (mean size, 48.0 mm); fifteen of them had enlarged lymph nodes in mediastina.

## Discussion

### Types and findings of primary trachea and main bronchus tumors

Primary trachea and main bronchus tumors have different findings due to different growth modes and development directions[23, 24]. There are three basic types of tumors according to their morphologies and relationship with walls. (1) Endoluminal nodular tumors with narrow bases: reveal as nodular masses and protrude into endolumen with narrow bases or peduncles connected to walls, no thickened walls, and homogeneous densities. In our series, 1 adenoma in trachea carina and 1 leiomyoma of left main bronchus had these findings. (2) Endoluminal nodular tumors with wide bases: manifest as nodular masses and protrude into endolumen with wide bases, locally thickened walls, and still homogeneous densities. One adenoid cystic carcinoma, 1 leiomyosarcoma, 2 adenocarcinomas and 4 squamous cell carcinomas of tracheae, 1 adenocarcinoma and 2 squamous cell carcinomas of right main bronchi, 1 mucoepidermoid carcinoma and 1 squamous cell carcinoma of left main bronchi, manifested as these findings in our series. (3) Intraluminal and extraluminal tumors: appear as infiltrated growth along thickened walls, invasion extraluminally, and masses both intraluminally and extraluminally. Tumors with dominant intraluminal growth have remarkable luminal stenoses. Tumors with obvious extraluminal growth may resemble mediastinum tumors. Tumors with apparent intraluminal and extraluminal growth have large masses, may with luminal obstruction entirely. In our series, 10 squamous cell carcinomas of tracheae, 3 squamous cell carcinomas of right main bronchi and 3 squamous cell carcinomas of left main bronchi belonged to this type.

### Differentiation of primary benign, malignant and low malignan tumors of tracheae and main bronchi

Benign tumors[23–25]: appear as small masses with diameter < 30.0 mm; round, oval, or polypiform, protrude into endolumen with narrow bases or peduncles, smooth and regular margins; no invasion of wall, fairly homogeneous wall thickness; homogeneous densities and not obvious enhancement; without enlarged lymph node in mediastinum. One adenoma in trachea carina and 1 leiomyoma of left main bronchus had these findings in our series.

Malignant tumors[24]: disclose as different sizes, usually > 30.0 mm in diameter; irregular or cayliflower, protrude into endolumen with wide bases and irregular luminal stenoses; invasion of walls, have intraluminal and extraluminal growth with thickened walls infiltratedly and heterogeneously; homogeneous or heterogeneous densities, obvious enhancements; usually with enlarged lymph nodes in mediastina. In our series, 14 squamous cell carcinomas, 2 adenocarcinomas and 1 leiomyosarcoma of tracheae, 5 squamous cell carcinomas and 1 adenocarcinoma of right main bronchi, 4 squamous cell carcinomas of left main bronchi displayed as these findings.

Low malignant tumors: their findings are between benign and malignant tumors. Show as polypiform masses, protrude into endolumen with fairly smooth and regular margins, homogeneous densities; but have comparatively wide bases, may grow along walls infiltratedly, or both intraluminally and extraluminally, with slightly thickened walls. One adenoid cystic carcinoma of trachea and 1 mucoepidermoid carcinoma of left main bronchus manifested as these findings in our series.

### Detection of primary trachea and main bronchus tumors with MDCT and its MPR, VR and VB postprocessing techniques

Though CT is the most important noninvasive method for detection of primary trachea and main bronchus tumors, ordinary CT and ordinary spiral CT can not disclose the entire structure of trachea and main bronchi and their detection is not accurate. MDCT is a new breakthrough in CT examination technique, can perform multislice data acquisition at the same time equipped with multidetector array, greatly reduce the time of volume scanning with obvious elevation of image resolution and quality, can obtain highly qualitative isotropic postprocessing images, and contribute to detecting primary trachea and main bronchus tumors.

MPR has two-dimensional axial, coronal, sagittal and oblique images with centering on trachea and main bronchus tumors, can reflect different density tissues by using different attenuation scales with high density resolution and not obvious artifact, clearly display lumen and longitudinal involvements of tumors and adjacent structures[7, 13–15]. Our results demonstrated that combination of axial, coronal, sagittal and oblique MPR images could not only display the locations, morphologies, internal features and extramural invasions of primary trachea and main bronchus tumors, morphologies and extents of luminal stenoses, but also measure their longitudinal involvements, distances between main bronchus tumors and trachea carinas; however, its images could not have three-dimensional effect due to two-dimensional images.

VR can use information from all the voxels in the data set, and the information is integrated into the resulting image, thus no information is lost, information on both depth and brightness are presented[11, 12]. VR is preferable to other postprocessing techniques such as shaded surface display (SSD), maximum intensity projection (MIP), minimum intensity projection (MinIP), and tissue transition projection (TTP), in which a large amount of data are lost in the final reconstructions[11, 12]. VR display as different bright, and regulate contrast resolution between different tissues according to demands; can retain the spatial relationship of initial data with less loss of image information, more anatomical level, and real three-dimensional effect; can manifest trachea and main bronchus luminal surface from outside to inside, and locally magnify and rotate images to disclose tumors as clearly as possible[16–18]. Our results proved that VR images could show locations, morphologies of primary trachea and main bronchus tumors, morphologies and extents of luminal stenoses, measure their longitudinal involvements and distances between main bronchus tumors and trachea carinas, but could not disclose their internal features and extramural invasions.

VB could allow three-dimensional visualization of the lumen and wall of the trachea and main bronchus. Its images are in a similar fashion to conventional optical bronchoscopy, and can display tumors in the same principle as virtual colonoscopy with direct showing of surface morphologies of tumors and proximal parts[12, 19–22]. Our study revealed that VB images could fairly display morphologies, longitudinal involvements of primary trachea and main bronchus tumors, morphologies and extents of luminal stenoses, but could not show their accurate locations, internal features, extramural invasions, and distances between main bronchus tumors and trachea carinae. Our study was consistent with that of Hoppe et al[12].

MDCT axial images can not accurately display morphologies, extramural invasions, longitudinal involvements of primary trachea and main bronchus tumors, morphologies and extents of luminal stenoses, distances between main bronchus tumors and trachea carinae; however, they are basic axial images, could show internal features of tumors, and contribute to detecting tumor natures[4, 5].

Therefore, MDCT and its MPR, VR and VB images have respective advantages and disadvantages. Their combination could complement to each other to disclose tumor locations, tumor morphologies, extramural invasions of tumors, longitudinal involvements of tumors, morphologies and extents of luminal stenoses, distances between main bronchus tumors and trachea carinae, and internal features of tumors; could detect trachea and main bronchus tumors from two-dimensional, three-dimensional and endoluminal structures; could greatly contribute to judging on benignancy, malignancy or low malignancy of primary trachea and main bronchus tumors with accurate detection of locations, natures and quantities, and provide crucial information for surgical treatment. Two benign, 2 low malignant and 27 malignant tumors in our series, MDCT combined with MPR, VR and VB not only accurately detected tumor locations, extramural invasions, benignancy, malignancy or low malignancy, but also determined longitudinal involvements, morphologies and extents of luminal stenoses, distances between main bronchus tumors and trachea carinae; their treatments were satisfactory with individual surgery program under the guidance of our detection information.

Our study has some limitations that should be taken into consideration. First, this retrospective study included a relatively small number of 31 consecutive patients. However, to our knowledge, it is the first series of evaluating the diagnostic value of MDCT and its MPR, VR and VB postprocessing techniques for primary trachea and main bronchus tumors. Second, 23 squamous cell carcinomas accounted for 74.2% of the 31 primary trachea and main bronchus tumors in our series; the rest tumors are adenocarcinomas (n = 3), leiomyoma (n = 1), leiomyosarcoma (n = 1), adenoma (n = 1), adenoid cystic carcinoma (n = 1), and mucoepidermoid carcinoma (n = 1), each type of tumor has only 1 or 3 cases, their numbers is small and their findings still require further investigation with more cases in the future.

In conclusion, MDCT and its MPR, VR and VB images have respective advantages and disadvantages. Their combination could complement to each other to accurately detect locations, natures (benignancy, malignancy or low malignancy), and quantities (extramural invasions, longitudinal involvements, extents of luminal stenoses, distances between main bronchus tumors and trachea carinae) of primary trachea and main bronchus tumors with crucial information for surgical treatment, are highly useful diagnostic methods for primary trachea and main bronchus tumors.

## Supporting Information

S1 Table Clinical data, MDCT and its MPR, VR and VB findings, surgery and pathology results. (XLS)
